# Diabetic Retinopathy in Patients with Diabetic Nephropathy: Development and Progression

**DOI:** 10.1371/journal.pone.0161897

**Published:** 2016-08-26

**Authors:** Chi-Juei Jeng, Yi-Ting Hsieh, Chung-May Yang, Chang-Hao Yang, Cheng-Li Lin, I-Jong Wang

**Affiliations:** 1 Department of Ophthalmology, National Taiwan University Hospital Hsin-Chu Branch, Hsinchu City, Taiwan; 2 Graduate Institute of Clinical Medicine, College of Medicine, National Taiwan University, Taipei, Taiwan; 3 Department of Ophthalmology, National Taiwan University Hospital, School of Medicine, Taipei, Taiwan; 4 Management Office for Health Data, China Medical University, Taichung, Taiwan; 5 Graduate Institute of Clinical Medical Science, China Medical University, Taichung, Taiwan; Roskamp Institute, UNITED STATES

## Abstract

The purpose of current study aims to investigate the development and progression of diabetic retinopathy (DR) in patients with diabetic nephropathy (DN) in a nationwide population-based cohort in Taiwan. Newly diagnosed DN patients and age- and sex-matched controls were identified from the Taiwanese Longitudinal Health Insurance Database from 2000 to 2010. We studied the effects of age, sex, hypertension, dyslipidemia, diabetic polyneuropathy (DPN), and medications on the development of nonproliferative DR (NPDR), proliferative DR (PDR), and diabetic macular edema (DME) in patients with DN. Cox proportional hazard regression analyses were used to estimate the adjusted hazard ratios (HRs) of the development of DR. Our results show that the adjusted HRs of NPDR and PDR were 5.01 (95% confidence interval (CI) = 4.68–5.37) and 9.7 (95% CI = 8.15–11.5), respectively, in patients with DN as compared with patients in the non-DN cohort. At 5-year follow-up, patients with DN showed an increased HR of NPDR progression to PDR (HR = 2.26, 95% CI = 1.68–3.03), and the major comorbidities were hypertension (HR = 1.23, 95% CI = 1.10–1.38 with NPDR; HR = 1.33, 95% CI = 1.02–1.72 with PDR) and DPN (HR = 2.03, 95% CI = 1.72–2.41 in NPDR; HR = 2.95, 95% CI = 2.16–4.03 in PDR). Dyslipidemia increased the HR of developing NPDR but not PDR or DME. Moreover, DN did not significantly affect DME development (HR = 1.47, 95% CI = 0.87–2.48) or progression (HR = 0.37, 95% CI = 0.11–1.20). We concluded that DN was an independent risk factor for DR development and progression; however, DN did not markedly affect DME development in this study, and the potential association between these disorders requires further investigation.

## Introduction

Diabetic retinopathy (DR) is the leading cause of blindness in working-age people [1]. As in the case of the global epidemic, diabetic retinopathy in Taiwan has been reported in 35% of all diabetic patients [2, 3]. In relation to the risk factors identified for DR, epidemiological studies conducted on both type 1 and type 2 diabetes mellitus (DM) patients from the Diabetes Control and Complications Trial (DCCT) and the Action to Control Cardiovascular Risk in Diabetes (ACCORD) Eye Study have revealed the importance of glycemic control in delaying or preventing DR development [4–6]. Moreover, disease duration, elevated blood pressure, lipid profiles, serum levels of advanced glycation end products (AGEs), evidence of early stage atherosclerosis, increased caliber of retinal blood vessels, and several genetic factors (such as those related to enzymes involved in glucose and lipid metabolism) also contribute to the development of DR [4].

Diabetic nephropathy (DN), the primary cause of chronic kidney disease, accounts for 40% of all new cases of end-stage renal disease development recorded annually [7]; DN is characterized by persistent albuminuria, progressive decline of glomerular filtration rate, and elevation of blood pressure [8, 9]. In patients with DN, the presence of albumin in urine not only signifies glomerular injury, but also reflects systemic endothelial abnormalities and vasculopathy, which can represent an independent risk factor for cardiovascular disease [10, 11]. As in the case of DR, the major risk factors identified for DN include prolonged duration of diabetes, poor glycemic control, and hypertension [12]. Furthermore, diabetic patients with proteinuria or on dialysis frequently present with vision-threatening DR and proliferative DR (PDR) [13] and are at risk for developing diabetic macular edema (DME) [14]. However, Man et al. reported, based on a cross-sectional study of 263 patients, that a reduction in glomerular filtration rate (eGFR) is associated with increased severity of DR, but not with DME [15]. Nevertheless, optimizing blood-sugar control together with tightly controlling blood pressure can reduce the risk of developing both DR and DN because the diseases share the same microvascular changes [16, 17].

In DR, chronic hyperglycemia causes endothelial damage, loss of pericytes, basement-membrane thickening, breakdown of the blood-retinal barrier (BRB), platelet aggregation, and leukocyte adhesion in retinal capillaries [18, 19]. The microstructure disarrangement and microcirculation dysfunction lead to vascular hyperpermeability and microaneurysm formation, as observed in nonproliferative DR (NPDR) [20, 21]. Excessive vascular leakage of fluids, proteins, or lipids in the macular area leads to the development of DME [22]. As the disease progresses, capillaries close and arterioles become atrophied, and this matches the nonperfusion areas detected in patients’ fluorescein angiography [23]. Eventually, chronic hypoxia induces the expression of several angiogenic growth factors, which results in retinal neovascularization, as observed in PDR [24, 25]. In DN, chronic hyperglycemia also alters the expression of growth factors and cytokines in renal glomeruli [26–29], and these changes, in turn, result in an imbalance of the hemodynamics in glomerular cells. In the early stages, glomerular hypertrophy and hyperfiltration occur as glomeruli respond to the expression of hyperglycemia. However, increased intraglomerular pressure and increased shear stress following loss of heparin sulfates in the glomeruli eventually lead to the thickening of the glomerular and tubular basement membrane, accumulation of the mesangial matrix, and albuminuria [30–32].

Given the findings of the aforementioned pathophysiological and epidemiological studies, we were intrigued by the association of vision-threatening DR, PDR, and DME with DN development according to the pathogenesis of the diseases, and to investigate the association, we conducted this population-based cohort study.

## Methods

### Data source

This study was conducted using the claim data obtained from the Longitudinal Health Insurance Database (LHID), which is a database of 1 million insurance claimants from the Taiwan National Health Insurance (Taiwan NHI) program. The Taiwan NHI was established in 1995 and it has served as a nationwide and compulsory health insurance program for Taiwan citizens. The National Health Research Institute (NHRI) established the LHID by randomly selecting 1 million insurance claimants from 1996 to 2000 and collecting their claim data annually. The LHID contains all of the data on claims from the Taiwan NHI, including the registry for beneficiaries, data on ambulatory care and hospital care claims, prescription files, and other medical expenditure files. The disease records in the Taiwan NHI were registered based on International Classification of Diseases, Ninth Revision, Clinical Modification (ICD-9-CM). To safeguard the privacy of claimants, the NHRI concealed the original identification numbers and provided scrambled and anonymous numbers before releasing the database. Moreover, this study was approved by the Ethics Review Board of China Medical University (CMUH104-REC2-115).

### Study population

In this study, we compared the risk of newly-diagnosed NPDR, PDR, and DME between DM patients with and without DN between January 1, 2000 and December 31, 2010. The DN cohort was composed of ≥18-year-old patients with DM (ICD-9-CM 250) plus DN (ICD-9-CM 249.4, 250.4), and the non-DN cohort was defined as the diagnosis of DN has not been made in this period. The non-DN cohort selected from the LHID comprised DM patients without a DN diagnosis and was 4-fold frequency matched by age and sex.

The outcomes of interest in this study were (1) NPDR (ICD-9-CM 250.5, 362.01, 362.03–06 362.1, 362.81, 362.82), (2) PDR (ICD-9-CM 362.02, 379.23) and administration of panretinal photocoagulation (PRP) treatment, and (3) DME (ICD-9-CM 362.53, 362.83, 362.07) and administration of IVI (intravitreal injection) treatment. The diagnosis of DME or the administration of IVI treatment rely on the results of OCT (ocular computer tomography) or FAG (fluorescein angiography) requested by the Taiwan National Health Insurance Program in insurance claimants on a reimbursement. Each patient included in the study was followed-up for each outcome. DR at the baseline of both DN and non-DN cohorts is excluded to determine the incidence of DR. The follow-up time was defined as the duration from the occurrence of NPDR, PDR, or DME to December 31, 2010. The diagnosis of NPDR, PDR, and DME was made at subsequent two visits with the same diagnosis. We also examined the 5-year risk of PDR and DME in the DN cohort and the occurrence of NPDR events in the non-DN cohort. These NPDR patients were followed-up until the patients withdrew from the health insurance program, the occurrence of PDR or DME events, or end of the 5-year follow-up.

We also investigated the influence of comorbidity and medication on the risk of NPDR, PDR, and DME. A patient with an identified comorbidity was defined as a patient with a history of the comorbidity before the index date; the comorbidities included were cerebrovascular accidents (CVA, ICD-9-CM 390–438), diabetic polyneuropathy (DPN, ICD-9-CM 357.2, 249.60, 249.61), hypertension (ICD-9-CM 401–405), and dyslipidemia (ICD-9-CM 272). The mediations considered were statin use, fibrate use, and antihypertension medication (including angiotensin-converting enzyme inhibitors, angiotensin II receptor blockers, α-blockers, β-blockers, calcium channel blockers, thiazides, and diuretics) before the index date.

### Statistical analysis

To compare DM patients with and without DN, we calculated the mean age and the corresponding standard deviation (SD) of the patients in the 2 cohorts and determined the number and percentages of males and females, the comorbidities, and the medications. The distribution difference between the study cohorts was assessed by performing *t* tests for age and the chi-square test for sex, comorbidities, and medications. The follow-up duration was calculated from the index date to the end of follow-up (person-years), and the incidence density was measured as the total number of events divided by the sum of the follow-up durations. The incidence curve for each cohort was also evaluated using the Kaplan-Meier method, and the differences in the curves were examined using the log-rank test. Moreover, the risk of NPDR, PDR, and DME in DM patients with DN was compared with the corresponding risk in the case of DM patients without DN; for this comparison, the hazard ratios (HRs) and 95% confidence intervals (CIs) were estimated using the crude and adjusted Cox proportional hazard models. We also compared the risk of NPDR, PDR, and DME between DM patients with and without DN after stratifying the patients according to age, sex, comorbidities, and medication. SAS 9.4 software (SAS Institute, Cary, NC, USA) was used to perform statistical analyses and R software was used to plot the incidence curve. The significant level was set at <0.05 for two-sided testing.

## Results

For this study, we included 10692 DM patients with DN and 42761 DM patients without DN (Table 1). The mean age of the patients in the 2 cohorts was approximately 64 years and nearly 50.5% of the study participants were aged ≥ 65 years old, and both cohorts included more males than females (53.3% vs 46.7%). The proportions of patients with hypertension, CVA, DPN, and dyslipidemia were significantly (all *P* < 0.001) higher in the DN cohort than in the non-DN cohort. Furthermore, statin use, fibrate use, and antihypertension medication were also more frequent in the DN cohort than in the non-DN cohort.

**Table 1:** Demographics and comorbidities of diabetes mellitus patients with and without diabetic nephropathy.

	Diabetic nephroropathy		
	No (N = 42761)	Yes (N = 10692)	
	n(%)	n(%)	*p*-value
Age, years			0.99
≤64	21156(49.5)	5289(49.5)	
≥65	21605(50.5)	5403(50.5)	
Mean (SD) ^†^	63.5(13.5)	64.0(13.4)	0.003
Gender			0.99
Female	19966(46.7)	4992(46.7)	
Male	22795(53.3)	5700(53.3)	
Comorbidity			
Hypertension	24771(57.9)	8279(77.4)	<0.001
CVA	27496(64.3)	8859(82.9)	<0.001
DPN	283(0.66)	378(3.54)	<0.001
Dyslipidemia	16783(39.3)	6463(60.5)	<0.001
Medication			
Statin	8180(19.1)	3848(36.0)	<0.001
Fibrate	6675(15.6)	3235(30.3)	<0.001
Antihypertensive medications	22331(52.2)	6945(65.0)	<0.001

Chi-square test was used to examine categorical data;

^†^*t* tests were used to examine continuous data;

CVA: cerebrovascular accidents

**Fig 1:**
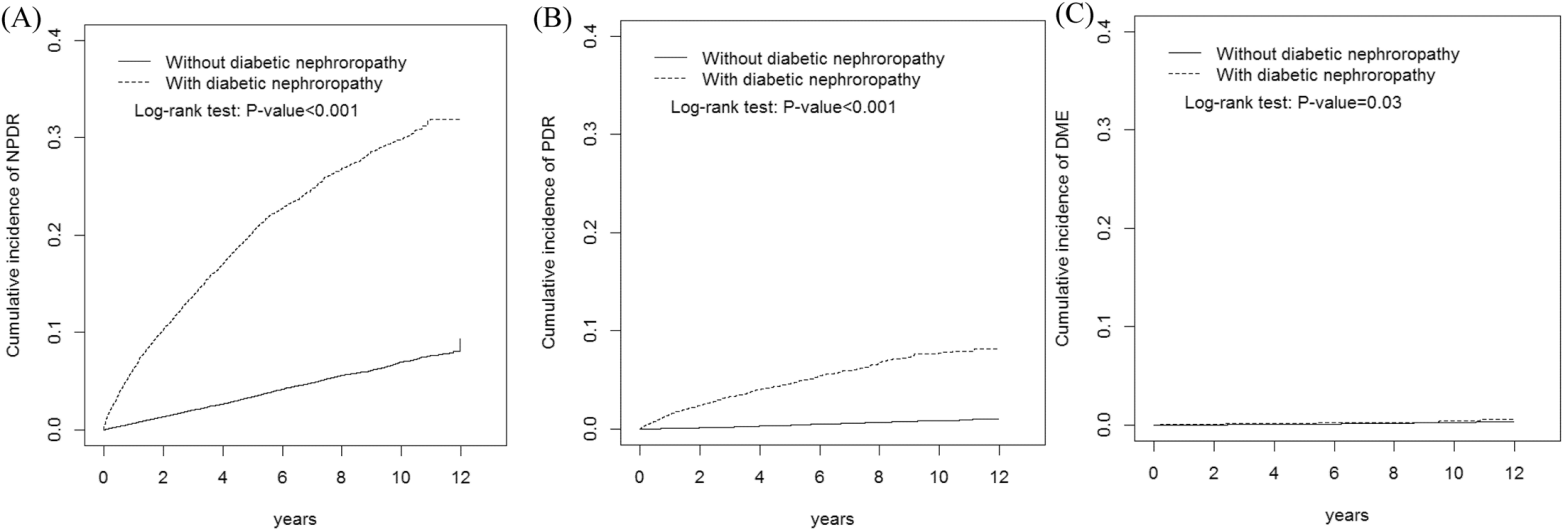
Cummulative incidences of (A) NPDR, (B) PDR, and (C) DME: comparison between diabetes mellitus patients with and without diabetic nephropathy.

The mean follow-up durations in the cases of the occurrence of NPDR, PDR, and DME were the following (respectively, in years): DN cohort, 4.26 (SD = 3.23), 4.91 (SD = 4.84), and 5.09 (SD = 3.27); non-DN cohort, 5.91 (SD = 3.27), 6.05 (SD = 6.02), and 6.06 (SD = 3.27). The cumulative incidence curves of NPDR (31.9% vs. 7.60%; P< 0.001), PDR (7.90% vs. 0.96%; P< 0.001), and DME (0.55% vs. 0.36%; P = 0.03) were plotted for the DN and non-DN cohorts (Fig 1), and the results revealed significantly higher numbers of NPDR, PDR, and DME events by the end of follow-up in the case of DM patients with DN than in the case of patients without DN. Moreover, we compared the risk of NPDR, PDR, and DME between the DN cohort and the non-DN cohort (Table 2). After adjustment for age, sex, comorbidity, and medication, the DM patients with DN showed, relative to patients without DN, nearly 5-fold higher risk of NPDR (HR = 5.01, 95% CI = 4.68–5.37) and 9.7-fold higher risk of PDR (HR = 9.70, 95% CI = 8.15–11.5); however, the risk of DME did not differ in a statistically significant manner between DM patients with and without DN (HR = 1.49, 95% CI = 0.88–2.51).

**Table 2:** Hazard ratios of outcomes according to sex, age, and comorbidity, obtained using univariate and multivariate Cox regression models.

	NPDR		PDR		DME	
Variable	Crude HR (95% CI)	Adjusted HR^†^ (95% CI)	Crude HR (95% CI)	Adjusted HR^†^ (95% CI)	Crude HR (95% CI)	Adjusted HR^†^ (95% CI)
Diabetic nephroropathy	5.90(5.53, 6.30)***	5.01(4.68, 5.37)***	11.1(9.42, 13.0)***	9.70(8.15, 11.5)***	1.74(1.06, 2.87)*	1.49(0.88, 2.51)
Gender (Women vs Men)	0.96(0.90, 1.02)	0.97(0.91, 1.04)	1.07(0.92, 1.24)	0.99(0.85, 1.15)	1.78(1.12, 2.81)*	1.95(1.23, 3.10)**
Age, years	1.00(0.99, 1.00)	0.99(0.99, 1.00)***	0.97(0.96, 0.97)***	0.96(0.96, 0.97)***	0.97(0.97, 0.98)***	1.02(1.00, 1.04)
Baseline comorbidities (yes vs no)						
Hypertension	1.77(1.65, 1.90)***	1.22(1.10, 1.37)***	1.43(1.22, 1.67)***	1.33(1.02, 1.73)*	2.30(1.39, 3.82)**	1.47(0.71, 3.04)
CVA	1.77(1.65, 1.91)***	1.11(0.99, 1.25)	1.39(1.18, 1.64)***	1.08(0.82,1.41)	2.565(1.48, 4.45)***	1.86(0.84, 4.10)
DPN	4.19(3.54, 4.96)***	2.03(1.71, 2.41)***	6.48(4.76, 8.82)***	2.95(2.16, 4.04)***	3.89(1.23, 12.3)*	3.00(0.93, 9.73)
Dyslipidemia	1.95(1.83, 2.09)***	1.21(1.12, 1.31)***	1.62(1.39, 1.88)***	0.86(0.71,1.03)	1.27(0.82, 1.98)	0.96(0.56,1.64)
Medication						
Statin	1.91(1.78, 2.06)***	1.17(1.08, 1.27)***	1.69(1.43, 2.00)***	1.09(0.90, 1.32)	2.08(1.27, 3.39)**	2.06(1.15, 3.69)*
Fibrate	1.76(1.63, 1.89)***	1.03(0.94, 1.12)	1.75(1.47, 2.07)***	1.12(0.92, 1.37)	0.86(0.45, 1.62)	0.56(0.28, 1.12)
Antihypertensive medications	1.44(1.35, 1.54)***	1.03(0.96, 1.12)	1.09(0.94, 1.26)	0.99(0.83, 1.18)	1.17(0.75, 1.82)	0.61(0.37, 1.01)

Crude HR: relative hazard ratio; Adjusted HR^†^: adjusted hazard ratio after controlling for age, sex, comorbidities (hypertension, CVA, DPN, and dyslipidemia), and medication (use of statin, fibrate, and antihypertension medication);

*P < 0.05,

**P < 0.01,

***P < 0.001.

Next, the risk of NPDR, PDR, and DME was compared between DM patients with and without DN after stratification by age, sex, comorbidity, and medication (Table 3). First, NPDR risk was significantly higher for the DN cohort than for the non-DN cohort following stratification by age, sex, comorbidity, and medication (all *P* < 0.001). The HR calculated for NPDR reached 9.21 (95% CI = 7.60–11.2) for the DN cohort as compared with non-DN cohort in the case of study patients without any comorbidity; however, the HR was only 4.82 (95% CI = 4.49–5.17) when we compared all patients presenting at least one comorbidity. Furthermore, the HR of NPDR was approximately 5-fold higher for the DN cohort than for the non-DN cohort in the case of participants who did not use any medication (HR = 5.75 for statin nonusers, 5.36 for fibrate nonusers, and 5.35 for antihypertension medication nonusers), and the HR was approximately 4-fold higher in the case of participants who used medications (HR = 3.83 for statin users, 4.38 for fibrate users, and 4.91 for antihypertension medication users). Second, the PDR risk calculated for the DN cohort was also higher than that determined for the non-DN cohort after stratification by age, sex, comorbidity, and medication (all *P* < 0.001). For the DM patients with DN, the HRs calculated for PDR (relative to patients without DN) were 12.3 (95% CI = 10.1–14.9) and 7.40 (95% CI = 5.27–10.4) in the case of statin nonusers and statin users, respectively. Third, although the overall DME risk did not differ in a statistically significant manner between DM patients with and without DN, in the younger age group (age ≤ 64), the risk of DME was significantly higher in the case of DM patients with DN than without DN (HR = 2.47, 95% CI = 1.21–5.05).

**Table 3:** Incidence and adjusted hazard ratio of NPDR, PDR, and DME according to sex, age, and comorbidity, compared between diabetes patients with and without diabetic nephropathy.

	Diabetic nephroropathy						Compared to Control	
	No			Yes				
Variables	Events n	PY	Rate^#^	Events n	PY	Rate^#^	Crude HR (95% CI)	Adjusted HR^†^ (95% CI)
NPDR								
All	1777	252522	7.04	1941	45547	42.6	5.90(5.53, 6.30)***	5.01(4.68, 5.37)***
Gender								
Female	877	122096	7.18	949	21672	43.8	5.95(5.43, 6.53)***	4.87(4.42, 5.37)***
Male	900	130427	6.90	992	23875	41.6	5.86(5.35, 6.42)***	5.11(4.65, 5.63)***
P for interaction								0.88
Age, years								
≤64	921	135413	6.80	1215	25929	46.9	6.74(6.19, 7.35)***	5.46(4.98, 5.99)***
≥65	856	117110	7.31	726	19619	37.0	4.89(4.43, 5.40)***	4.44(4.01, 4.93)***
P for interaction								<0.001
Comorbidity^§^								
No	336	74515	4.51	158	3984	39.7	8.83(7.31, 10.7)***	9.21(7.60, 11.2)***
Yes	1441	178007	8.10	1783	41563	42.9	5.17(4.82, 5.54)***	4.82(4.49, 5.17)***
P for interaction								<0.001
Medication								
Statin								
No	1343	215081	6.24	1276	31405	40.6	6.41(5.94, 6.92)***	5.74(5.30, 6.23)***
Yes	434	37441	11.6	665	14143	47.0	3.96(3.51, 4.47)***	3.81(3.37, 4.31)***
P for interaction								<0.001
Fibrate								
No	1437	218233	6.58	1337	32559	41.1	6.11(5.67, 6.58)***	5.36(4.95, 5.79)***
Yes	340	34290	9.92	604	12989	46.5	4.58(4.01, 5.23)***	4.38(3.82, 5.01)***
P for interaction								<0.001
Antihypertensive medications								
No	806	132605	6.08	704	17435	40.4	6.56(5.93, 7.26)***	5.35(4.79, 5.97)***
Yes	971	119917	8.10	1237	28113	44.0	5.29(4.86, 5.76)***	4.91(4.50, 5.36)***
P for interaction								0.003
PDR								
All	209	258959	0.81	478	52476	9.11	11.1(9.42, 13.0)***	9.70(8.15, 11.5)***
Gender								
Female	100	125145	0.80	219	25355	8.64	10.6(8.37, 13.4)***	8.69(6.74, 11.2)***
Male	109	133451	0.82	259	27121	9.55	11.5(9.21, 14.4)***	10.6(8.36, 13.5)***
P for interaction								0.64
Age, years								
≤64	155	138406	1.12	385	30084	12.8	11.2(9.33, 13.6)***	10.2(8.35, 12.4)***
≥65	54	120189	0.45	93	22391	4.15	8.96(6.40, 12.5)***	9.19(6.43, 13.1)***
P for interaction								0.24
Comorbidity^§^								
No	53	75606	0.70	47	4599	10.2	14.6(9.86, 21.6)***	13.5(9.03, 20.1)***
Yes	156	182989	0.85	431	47877	9.00	10.4(8.65, 12.5)***	8.99(7.45, 10.8)***
P for interaction								0.14
Medication								
Statin								
No	165	219790	0.75	332	36024	9.22	12.2(10.1, 14.7)***	12.3(10.1, 14.9)***
Yes	44	38805	1.13	146	16452	8.87	7.73(5.52, 10.8)***	7.40(5.27, 10.4)***
P for interaction								0.02
Fibrate								
No	177	223163	0.79	333	37422	8.90	11.0(9.19, 13.2)***	11.0(9.10, 13.4)***
Yes	32	35432	0.90	145	15054	9.63	10.6(7.22, 15.5)***	10.4(7.08, 15.3)***
P for interaction								0.83
Antihypertensive medications								
No	117	135290	0.86	206	19867	10.4	11.8(9.38, 14.8)***	10.9(8.50, 13.8)***
Yes	92	123305	0.75	272	32608	8.34	11.1(8.72, 14.0)***	10.8(8.48, 13.8)***
P for interaction								0.71
DME								
All	59	259142	0.23	21	54420	0.39	1.74(1.06, 2.87)*	1.49(0.88, 2.51)
Gender								
Female	22	125471	0.18	6	26302	0.23	1.38(0.56, 3.39)	1.21(0.46, 3.16)
Male	37	133672	0.28	15	28119	0.53	1.96(1.07, 3.57)*	1.61(0.86, 3.01)
P for interaction								0.48
Age, years								
≤64	20	138870	0.14	16	31692	0.50	3.58(1.85, 6.90)***	2.49(1.22, 5.10)*
≥65	39	120273	0.32	5	22729	0.22	0.71(0.28, 1.81)	0.68(0.27, 1.76)
P for interaction								0.006
Comorbidity^§^								
No	8	75760	0.11	0	4811	0.00	-	-
Yes	51	183382	0.28	21	49609	0.42	1.55(0.93, 2.57)	1.58(0.94, 2.65)
P for interaction								0.98
Medication								
Statin								
No	44	220220	0.20	13	37439	0.35	1.76(0.95, 3.27)	1.61(0.85, 3.06)
Yes	15	38922	0.39	8	16982	0.47	1.25(0.53, 2.94)	1.17(0.48, 2.85)
P for interaction								0.51
Fibrate								
No	54	223640	0.24	15	38754	0.39	1.64(0.92, 2.90)	1.15(0.64, 2.09)
Yes	5	35502	0.14	6	15665	0.38	2.75(0.84, 9.00)	2.95(0.88, 9.84)
P for interaction								0.44
Antihypertensive medications								
No	29	135583	0.21	9	20678	0.44	2.08(0.98, 4.39)	1.41(0.63, 3.14)
Yes	30	123560	0.24	12	33742	0.36	1.49(0.76, 2.91)	1.41(0.71, 2.81)
P for interaction								0.49

PY: person-years; Rate^#^: incidence rate, per 1000 PY; Crude HR: relative hazard ratio; Adjusted HR^†^: adjusted hazard ratio after controlling for age, sex, comorbidities (hypertension, CVA, DPN, and dyslipidemia), and medication (use of statin, fibrate, and antihypertension medication); Comorbidity^§^: the comorbidity group included patients with any one of these comorbidities: hypertension, CVA, DPN, and dyslipidemia;

*P < 0.05,

**P < 0.01,

***P < 0.001

Lastly, we compared the risk of PDR and DME between DM patients with and without DN after the occurrence of NPDR during the 5-year follow-up period (Table 4). After NPDR occurrence, PDR risk was significantly higher in the case of DM patients with DN than in the case of DM patients without DN (HR = 2.25, 95% CI = 1.68–3.02), but DME risk did not differ significantly between the 2 groups (HR = 0.37, 95% CI = 0.12–1.22).

**Table 4:** Overall PDR and DME events and hazard ratios of PDR and DME measured for NPDR among study participants within a 5-year follow-up period.

	NPDR						Compared to Control	
	No			Yes				
Variables	Events n	PY	Rate^#^	Events n	PY	Rate^#^	Crude HR (95% CI)	Adjusted HR^†^ (95% CI)
PDR	66	5316	12.4	177	5892	30.0	2.48(1.87, 3.30)***	2.25(1.68, 3.02)***
DME	11	5446	2.02	4	6394	0.63	0.33(0.11, 1.04)	0.37(0.12, 1.22)

PY: person-years; Rate^#^: incidence rate, per 1000 PY; Crude HR: relative hazard ratio; Adjusted HR^†^: adjusted hazard ratio after controlling for age, sex, comorbidities (hypertension, CVA, DPN, and dyslipidemia), and medication (use of statin, fibrate, and antihypertension medication);

***P < 0.001.

## Discussion

DR, DN, and DPN are the most common complications related to small-vessel injuries due to long-term hyperglycemia [33, 34]. Previously, we reported that DPN and DR were correlated: patients with DPN presented an elevated risk of developing DR and PDR [35]. Similarly, Barr et al. reported a correlation between these 3 microvascular complications and indicated that patients with DPN exhibit (relative to control) a 4-fold increase in DR rate and a 2-fold increase in the rate of microalbuminuria [36]. Here, we further demonstrated that the incidences of NPDR, PDR, and DME increased with time in patients with DN, who also presented a higher rate to DR development than did patients without DN (Fig 1). We also demonstrated that relative to patients without DN, patients with DN carried a higher risk of developing NPDR and PDR and progression from NPDR to PDR during the 5-year follow-up (Tables 3 and 4), which agrees with the results of Wisconsin Epidemiologic Study of Diabetic Retinopathy (WESDR) [8, 9]. Intriguingly, the effect of DN on NPDR development was measured to be markedly elevated after we stratified and adjusted the effects of other risk factors and comorbidities in this study. In the case of patients without any comorbidity, DN increased the risk of NPDR development 9.21 fold, whereas the increase was 4.82 fold in patients presenting other comorbidities. The patients with DN also carried an elevated risk of developing PDR, although the independent effect of DN did not differ in a statistically significant manner between patients with and without comorbidities. In the longitudinal 5-year follow-up, DN was also identified to increase the risk of NPDR progression to PDR. However, the effect on DME development was not statistically significant. These findings partly agree with the risk factors for DR occurrence and progression that were reported based on WESDR [8, 9] and the UK Prospective Diabetes Study (UKPDS) [21], which demonstrated that the duration of diabetes, degree of metabolic control, elevated glycosylated hemoglobin A1c levels, severity of DR, hypertension, low socioeconomic status, and older age are DME risk factors [37, 38].

Regarding the duration of DM, our study cannot provide the duration of DM in this health claim database. However, in the Table 1, the difference of the age between DN cohort and non-DN cohort was statistically insignificant. In the Table 2, there was no effect of age on the development of NPDR, PDR and DME. The mean follow-up durations in the cases of the occurrence of NPDR, PDR, and DME were the following: DN cohort, 4.26 years, 4.91 years, and 5.09 years; non-DN cohort, 5.91 years, 6.05 years, and 6.06 years. In spite of the absence of duration of DM in this study, our results revealed that the presence of DN has a significant role in the development of NPDR or PDR, but the effect of age was insignificant. Nevertheless, the duration of diabetes may still have a major impact on DR rates and be a confounder. This limitation should be investigated in future studies.

We determined that hypertension was associated in a statistically significant manner with the development of NPDR and PDR but not DME (Table 2); this result is partly in accord with the results of previous studies indicating that increased systolic blood pressure is a major risk factor for DR [39]. Although certain cross-sectional data have suggested that hypertension is associated with DR, longitudinal data have been inconsistent [40–45]. The UKPDS results showed that DR incidence was associated with systolic blood pressure [46], and in WESDR, diastolic blood pressure was identified as a statistically significant independent predictor of DR progression to PDR over a 14-year follow-up period in patients with younger-onset (type 1) DM, regardless of glucose control and proteinuria [47]; however, no association was identified in the case of type 2 DM, which might be due to the original selection of older-onset diabetes and mortality rates [47]. By contrast, in another study, diastolic blood pressure in the fourth-quartile range was identified to be associated with a 3.3-fold increase in the 4-year risk of developing DME as compared with the blood pressure in the first-quartile range in patients with younger-onset DM, and further with a 2.1-fold increase in the risk in the case of patients with older-onset diabetes [48]. Moreover, the results of a randomized clinical trial demonstrated that a lowering blood pressure to below 140/90 mmHg was associated with a substantial reduction in the risk of developing macrovascular and microvascular complications in hypertensive patients with DM [49]. In this study, DN patients with hypertension presented a higher risk of developing NPDR and PDR than did DN patients without hypertension. Medical control of blood pressure exerts a protective effect in the early but not late stage of DR, which agrees with the findings of certain studies showing that blood pressure control might not be able to halt disease progression to the proliferative stage or macular edema development [50]. However, another study showed that the use of an angiotensin-converting enzyme inhibitor for blood pressure control might protect against DR progression [51].

The possible pathogenic mechanisms by which hypertension affects DR are (1) hemodynamic mechanisms (impaired autoregulation and hyperperfusion) and (2) vascular endothelial growth factor (VEGF)-dependent mechanisms, because hypertension independent of hyperglycemia upregulates VEGF expression in retinal endothelial cells and ocular fluids [52]. Therefore, we conclude that DR duration, hypertension, hypertension treatment, and potential ethnic factors led to a nonsignificant effect of hypertension on DME development in this study.

CVA was identified here as a comorbidity in the cohort of patients with DN (Table 1), although CVA alone did not increase the HRs of NPDR, PDR, and DME (Table 2). Furthermore, the interactions among hypertension, CVA, dyslipidemia, and DPN increased the adjusted HRs of developing NPDR and PDR, but not DME, in patients with DN (Table 3). Our findings agree with the results of previous studies showing that diabetic patients with DN carry an increased risk of fatal and nonfatal cardiovascular and other complications [53–57], and this risk is also affected by genetic and ethnic predisposition [58, 59]. However, the most notable difference in the case of our results is that these comorbidities did not contribute to the development of DME in the DN cohort. Although the association between DR and cardiovascular outcomes has been extensively studied [60–63] and reviewed [64], the cardiovascular outcomes in DME patients remain inadequately examined; in previously studies that included DME patients, the statistical power was insufficient to characterize the relationship between DR and cardiovascular outcomes [62, 63]. For example, in one study, a large insured population was used to quantify and compare the incidence rates of myocardial infarction or CVA/stroke in hospitalized patients with DME against matched diabetic control patients [65]. The adjusted rate ratio calculated for CVA was 1.98 (95% CI: 1.39–2.83, *P* < 0.001) for DME patients versus the diabetic controls. By contrast, our cohorts included hospitalized and nonhospitalized patients, and this is likely to be comparatively more representative of patients with DME.

With regard to dyslipidemia, our results showed that patients under statin and fibrate medication developed DN more frequently than did patients who did not receive these treatments (Table 1). Moreover, the hazard ratio calculated for NPDR, PDR, and DME were higher in patients who received statins than in patients who received fibrate (Table 2). Similarly, statin and fibrate use in DN patients increased the adjusted HRs of developing NPDR and PDR but not DME (Table 3). These results indicate that dyslipidemia plays a role in the development of DR and DN, and that statin and fibrate use can lower the risk of developing DME in patients with dyslipidemia. Serum lipids have been reported to be a risk factor for DR and DME [66, 67], and permeability changes in the retinal microvasculature have been suggested to result in extravascular accumulation of lipoprotein deposits coupled with a consequent loss of function in the surrounding retinal cells [68, 69]; however, the role of serum lipids in the pathogenesis of DR and DME remains controversial. The Fenofibrate Intervention and Event Lowering in Diabetes study reported that fenofibrate treatment resulted in reduced DR progression and a diminished requirement for laser treatment in type 2 DM in the study participants [70]. The Action to Control Cardiovascular Risk in Diabetes Eye study showed that concomitant use of fenofibrate and statin reduced the requirement for laser treatment by 40% [71], which is compatible with the treatment’s protective effect against NPDR development in DN patients that was observed in this study. However, these findings suggest a complex mechanistic association between serum dyslipidemia and DR and DME, the underlying pathogenetic process of which remains unclear. Although evidence gathered from cohort studies and meta-analyses of case-control studies have suggested a strong relationship between lipid levels and DME, this relationship was not confirmed by a meta-analysis that included only prospective random clinical trials [72]. Thus, the relationship between lipid levels and DME warrants further investigation.

The most intriguing result obtained in this study was that the risk of DME development did not differ between patients with and without DN, although patients with DN still showed an increased the risk of developing PDR from NPDR than did the patients without DN. In patients aged less than 64 years old, DN can influence DME development to a certain extent. As mentioned, DME is a complex disease of multifactorial origin that is caused by a disruption of the BRB [73]. Chronic elevation of blood glucose, high cholesterol, accumulation of oxygen free radicals and AGEs/AGE receptors, protein kinase C, and other factors have been implicated in the pathogenesis of DME [22]. These factors ultimately contribute to an increase in VEGF expression, which results in a breakdown of the BRB. Moreover, although reversible, hyperglycemia impairs the function of the retinal pigment epithelium at an early stage of the disease [74]. In addition to the increased permeability of retinal capillaries, the primary retinal change in DR, the closure of retinal capillaries leads to retinal ischemia. Retinal ischemia, in turn, can result in the formation of neovascularization, which might lead to vitreous hemorrhage or traction damage in the retina through the production of various growth factors, including VEGF [75].

In the pathogenesis of DN, as in DME pathogenesis, podocytes secrete increased amounts of VEGF-A [76], tight-junction loss occurs and leads to hyperpermeability, and albuminuria is prevalent [77, 78]. As the nephropathy progresses, DN is eventually associated with capillary nonperfusion, which leads to podocyte death in DN and to increased extracellular matrix deposition and thus a thickening of the glomerular basement membrane [79, 80], as in PDR [81]. These findings could explain the results of our study, which demonstrated that young patients with DN or patients presenting the early events of DN carried an elevated risk of DME, which coexisted with capillary hyperpermeability and the presence of albuminuria. However, these parallel and intercorrelated diseases progressed together, and the DM patients with DN presented an increased risk of PDR in the long-term follow-up.

In summary, our findings indicate that patients with DN experience higher incidences of DR and progression to PDR as compared with patients without DN. Moreover, the results confirmed that follow-up duration and hypertension are associated with DR development, and that lipid-modulating agents exert a protective effect during the early stages of DR. Patients with type 2 DM and albuminuria must be carefully monitored for progressive eye disease, and patients with DME must be evaluated for concomitant kidney disease. DME formation (which might be multipathogenic) and its correlation with DN require further investigation.
